# At-Risk Serum Cholesterol Profile at Both Ends of the Nutrition Spectrum in West African Adults? The Benin Study

**DOI:** 10.3390/nu5041366

**Published:** 2013-04-19

**Authors:** Hélène Delisle, Gervais Ntandou, Roger Sodjinou, Charles Couillard, Jean-Pierre Després

**Affiliations:** 1 TRANSNUT, WHO Collaborating Centre on Nutrition Changes and Development, Department of Nutrition, Faculty of Medicine, University of Montreal, PO Box 6128, Downtown Station, Montreal, QC H3C 3J7, Canada; 2 TRANSNUT, Department of Nutrition, University of Montreal, PO Box 6128, Downtown Station, Montreal, QC H3C 3J7, Canada; 3 Bioversity International, c/o IITA, 08 BP 0932 Cotonou, Benin; E-Mail: g.ntandou-bouzitou@cgiar.org; 4 UNICEF-Chad, BP 1146, N’Djaména, Chad; E-Mail: rsodjinou@unicef.org; 5 Institute of Nutraceuticals and Functional Foods, Department of Food Science and Nutrition, Laval University, Quebec City, G1V 0A6, Canada; E-Mail: charles.couillard@fsaa.ulaval.ca; 6 University Institute of Cardiology and Pneumology and Division of Kinesiology, Department of Social and Preventive Medicine, Laval University, Quebec City, G1V 0A6, Canada; E-Mail: jean-pierre.despres@criucpq.ulaval.ca

**Keywords:** HDL-cholesterol, West Africa, metabolic syndrome, dietary quality, nutritional status, double burden of malnutrition, lifestyle, nutrition transition

## Abstract

Low HDL-cholesterol (HDL-C), using as cut-offs 1.03 mmol/L in men and 1.29 mmol/L in women, was observed in more than 25% apparently healthy adults (*n* = 541) in a cross-sectional study on nutrition transition and cardiometabolic risk factors (CMRF) in Benin, West Africa. Both overweight/obesity (35.3%) and underweight (11.3%) were present, displaying the double burden of malnutrition. We examined in more depth the association of low HDL-C with nutrition and with other CMRF. Metabolic syndrome components were assessed, plus the ratio of total cholesterol (TC)/HDL-C and serum homocysteine. Insulin resistance was based on Homeostasis Model Assessment. We also measured BMI and body composition by bio-impedance. Dietary quality was appraised with two non-consecutive 24 h recalls. Low HDL-C was associated with much higher TC/HDL-C and more abdominal obesity in men and women and with more insulin resistance in women. The rate of low HDL-C was highest (41.9%) among the overweight/obese subjects (BMI ≥ 25), but it also reached 31.1% among the underweight (BMI < 18.5), compared with 17.3% among normal-weight subjects (*p* < 0.001). Lower dietary micronutrient adequacy, in particular, in vitamins A, B_3_, B_12,_ zinc and calcium, was associated with low HDL-C when controlling for several confounders. This suggests that at-risk lipoprotein cholesterol may be associated with either underweight or overweight/obesity and with poor micronutrient intake.

## 1. Introduction

Low income countries undergoing a rapid transition towards Western dietary patterns and sedentary lifestyles [1] are now facing the double burden of malnutrition, that is, the coexistence in the same population of “undernutrition” and “overnutrition” [2]. Undernutrition encompasses general and specific nutritional deficiencies, while overnutrition refers to obesity and other nutrition-related non-communicable diseases (NCDs). NCDs are indeed a growing concern in developing countries, and WHO has invited countries to invest in the prevention of obesity, diabetes, cardiovascular disease (CVD) and some types of cancer [3]. Priority actions have been proposed at the NCD Summit of 2011 [4].

Dyslipidaemia is a major risk factor for CVD across population groups, as was shown in the INTERHEART Study [5]. It is part of the metabolic syndrome (MetS), an aggregation of cardio-metabolic risk factors (CMRF) that include insulin resistance, hyperglycemia, high blood pressure and abdominal obesity [6]. In the MetS, dyslipidaemia is defined as high triglyceride concentrations or low HDL-cholesterol (HDL-C). Low HDL-C is a well-established and independent CMRF [7]. The antiatherogenic properties of HDL-C are related to its role in reverse cholesterol transport, endothelial cell function regulation and inhibition of oxidation, thrombosis and inflammation through complex intracellular signalling pathways [7,8]. In diabetic subjects, low HDL-C is an independent risk factor for the development of macrovascular, as well as microvascular complications [9].

Low HDL-C is associated with increasing risk of CVD with advancing age, perhaps even more so among women, and weight gain appears to be the main culprit of low HDL-C [10,11]. A high prevalence of low HDL-C among adults has been reported as the most prevalent lipid abnormality in Mexicans [12]. Turks and other Asians, including Bangladeshi and Pakistani populations, have among the highest prevalence rates of low HDL-C worldwide [13]. In African and African-descent populations, total cholesterol and triglycerides are usually lower than in Caucasians, with an associated lower coronary risk. However, low HDL-C is a common feature of MetS in these populations [14,15,16,17]. While genetic factors are major determinants of HDL-C, environmental factors, such as alcohol consumption, tobacco, physical activity, diet and obesity are also at play [18]. Interestingly, vegetarians, whose intake of saturated and unsaturated fat is limited, have low HDL-C, but a low cardiovascular risk [18]. The rather high occurrence of low HDL-C in Africans and other developing country populations might be associated with low fat intake, other dietary inadequacies and undernutrition, but this is not well documented in spite of its relevance for countries facing the double burden of malnutrition.

In the Benin study, we indeed observed that low HDL-C was highly prevalent, while hypertriglyceridaemia and high low-density lipoprotein cholesterol (LDL-C) were uncommon [19,20]. The rate of low HDL-C (<1.03 in men; <1.29 in women [6]) was 22.5% in men and 32.6% in women. Low HDL-C was the MetS component exhibiting the highest prevalence in this population. Similar findings were also reported in apparently healthy adults of Ouagadougou, the capital city of Burkina Faso, with 28% of men and 32% of women having low HDL-C [21]. Examining this intriguing feature of the MetS in Benin was therefore deemed relevant. Additionally, in the Benin study, 12.5% of men and 10% of women were underweight, whereas 19.9% of men and 50.7% of women were overweight, including 4.4% obese men and 22.2% of obese women. This was an opportunity to examine nutritional determinants and cardiometabolic correlates of low HDL-C in more depth, in this context of the double burden of malnutrition. The working hypothesis was that low HDL-C would also occur in undernutrition and not only overnutrition.

“Overnutrition” is somewhat a misnomer, because dietary imbalance and not only excess may be associated with NCDs. Malnutrition is generally perceived as a synonym for undernutrition, and it would seldom be equated with “overnutrition”. For this reason, the term “dysnutrition” may be more relevant than “malnutrition” to encompass the whole range of nutritional disorders, from general undernutrition or specific micronutrient deficiencies, to diet-related NCDs [22]. “Dysnutrition” is not as yet accepted, however. In the present paper, undernutrition and overnutrition refer to either anthropometric status or dietary intakes, whereas underweight and overweight/obesity refer exclusively to anthropometric status.

## 2. Experimental Section

### 2.1. Subjects and Study Design

This cross-sectional Benin study was conducted in Cotonou, the largest city in the country, in Ouidah, a small-size city located 50 km away from Cotonou (semi-urban area) and in the rural area surrounding Ouidah. Subjects (*n* = 541) were randomly selected by multistage sampling in the urban (*n* = 200), semi-urban (*n* = 171) and rural (*n* = 170) areas, using census data (urban) or after enumeration of compounds (semi-urban and rural). Eligible participants were Beninese-born adults aged 25 to 60, having lived in the study area for at least six months. Subjects with a prior diagnosis of hypertension, diabetes or cardiac condition were excluded, because of possible modification of their diet and lifestyle, but the cases detected after the study began were retained in data analyses. As dyslipidemia is not diagnosed as such, it was not an exclusion criterion, as it would unlikely affect people’s diet. Data were collected between 2006 and 2008. Details of study protocol may be found elsewhere [19,23,24,25].

The study was approved by the Ethics Committee of the Faculty of Medicine, University of Montreal, and by the Ministry of Health in Benin.

### 2.2. Biological and Biochemical Variables

Anthropometric variables included body weight, height and waist circumference measured according to standard procedures. BMI cut-offs for underweight (<18.5), overweight (25–29.9) and obesity (≥30) were as defined by WHO [26]. Waist circumference (WC) was measured as index of abdominal obesity [27]. Fat mass and fat-free mass (FFM) were computed by bioelectric impedance using sex and ethnicity-specific equations [28]. Blood pressure was measured as described previously [23]. Blood samples were collected after a 12-h overnight fast and were stored in ice boxes until centrifugation within two hours. Fasting plasma glucose and serum concentrations of total cholesterol (TC), LDL-C, HDL-C and triglycerides were determined using standard laboratory methods. The ratio of TC/HDL-C was computed. MetS components were as defined in the joint statement of International Diabetes Federation (IDF) taskforce [6]: abdominal obesity (WC ≥ 80 cm in women and ≥94 cm in men); elevated triglycerides (>1.70 mmol/L); high blood pressure (≥130/85 mmHg); high fasting glucose (≥5.6 mmol/L); and low HDL-C (≤1.29 mmol/L in women and ≤1.03 mmol/L in men). The insulin resistance cut-off was the seventy-fifth centile of Homeostasis Model Assessment (HOMA) in the whole sample of Benin subjects, as advocated [29]. HOMA at the seventy-fifth centile in the Benin study was 3.2. The selected cut-off for high TC/HDL-C was 4 for men and 5 for women [30]. Serum homocysteine was determined with ELISA (Axis^®^ Homocysteine, Hamburg, Germany), and the cut-off was 12 µmol/L [31].

### 2.3. Socio-Economic and Demographic Variables

Socio-economic and demographic data were collected in individual interviews. The level of education was assessed. Socio-economic status (SES) was based on a household amenity score as proxy for income, as used in several Demographic and Health Surveys in Africa. The items include type of housing, material possessions and utilities. The SES score was computed separately in each location and tertiles were used in the analyses. Details of items and coding are available elsewhere [19,23,24,25].

### 2.4. Dietary Quality

Dietary intake was computed on the basis of two or three non-consecutive 24-h food recalls conducted over an average period of one month. Diet quality was appraised with a micronutrient adequacy score and a preventive diet score developed by our group for international use. The micronutrient score was based on adequacy of intake of 14 micronutrients (vitamins A, B_6_, B_12_, C and E, thiamine, riboflavin, niacin, pantothenic acid, folate, magnesium, calcium, iron and zinc) according to WHO/FAO recommended dietary intakes for age and sex [32]. The preventive diet score was based on compliance with eight WHO/FAO dietary guidelines for the prevention of chronic diseases (% total fat, % saturated fatty acids, % polyunsaturated fatty acids, cholesterol (mg/day), % sugar, % protein, fiber (g/day), as well as fruits and vegetables (g/day)) [33]. For each item, a rating of “1” was given if the intake met the recommendation and “0” if it did not. Further details on dietary methods were previously published [25].

### 2.5. Alcohol Consumption and Smoking

Subjects were asked about their usual drinking patterns. An alcohol consumption score was based on the pattern of alcohol consumption and the mean quantity of alcohol consumed daily. Three categories were identified: non-drinkers, moderate alcohol consumption and heavy drinkers. Smoking habits were defined as: current smokers, ex-smokers and non-smokers. For alcohol and tobacco, the questionnaire was based on the STEPwise tool developed by WHO [34].

### 2.6. Physical Activity

Physical activity was assessed through three non-consecutive 24-h recalls [24]. Participants were asked about all their previous day activities, from the time they got up to the moment they went to bed. Time spent in bed, in various modes of transportation, for main and secondary occupations, for house chores and for leisure activities was computed from the daily estimated schedule. The intensity level of each activity was estimated in metabolic equivalents (MET). Based on WHO guidelines for the prevention of chronic diseases [33], we described subjects as active (≥3 MET and ≥30 min/day) and inactive (<30 min/day or <3 MET).

### 2.7. Statistical Analyses

Data were analyzed using IBM SPSS, version 19.0. The independent contribution of lifestyle and dietary factors to HDL-C was assessed in multivariate regression models controlling for socio-economic and demographic variables. The interaction of sex by zone was examined to verify whether the association of sex with HDL-C was modulated by the rural, semi-urban or urban location. Univariate statistics used as appropriate *t-*tests and chi^2^ with odds ratios (ORs). ORs of cardio-metabolic risk factors in low HDL-C compared to normal HDL-C were computed. The level of statistical significance was a *p* value of <0.05.

## 3. Results

The data on the 541 subjects (50% women) of the Benin study were used in the analyses. The results are broken down according to HDL-C status.

### 3.1. Socio-Economic Characteristics

As seen in Table 1, residence area was significantly associated with HDL-C, with a significantly lower rate of low HDL-C in the semi-urban group compared to the rural and the urban groups. Age, education and SES were unrelated to HDL-C status in these univariate analyses.

**Table 1:** Subjects’ characteristics according to high-density lipoprotein cholesterol (HDL-C) level.

	(*n*)	Low HDL-C (%)	Normal HDL-C (%)	*p* ^1^
*Gender*				<0.01
Women	(270)	32.6	67.4	
Men	(271)	22.5	77.5	
All	(541)	27.5	72.5	
*Age category*				0.78
25–34 years	(209)	27.8	72.2	
35–44 years	(225)	26.2	73.8	
45 years+	(107)	29.9	70.1	
*Residence area*				<0.001
Rural	(170)	25.3	74.7	
Semi-urban	(171)	18.1	81.9	
Urban (Cotonou)	(200)	37.5	62.5	
*Education*				0.39
None	(138)	28.3	71.7	
Primary school	(187)	24.1	75.9	
High school	(216)	30.1	69.9	
*SES score tertile*				0.25
Low	(186)	23.1	76.9	
Middle	(195)	29.7	70.3	
High	(160)	30.0	70.0	

^1^ Chi^2^.

### 3.2. Serum Lipid and Body Composition

Low HDL-C was associated in men and women with significantly higher values for TC/HDL-C, BMI and WC (Table 2). In both men and women, LDL-C was normal and showed no association with HDL-C level. In women only, low HDL-C was also associated with significantly higher triglyceride concentrations, higher fat-free body mass and higher body fat mass. Table 2 also shows than mean BMI was normal in men, but elevated in women.

### 3.3. Nutritional (Anthropometric) Status, Diet Quality and Lifestyle

BMI and WC status, dietary quality scores and lifestyle patterns are presented in Table 3 according to HDL-C level, for men and women. Anthropometric status was significantly associated with HDL-C in men and women. Overweight/obesity and abdominal obesity were much more widespread in women than men. Not only were overweight/obesity and abdominal obesity associated with low HDL-C, but so was underweight. Overall, the rate of low HDL-C was highest among the overweight/obese (41.9% of the 191 overweight-obese subjects), but it also reached 31.1% of the 61 underweight subjects, compared with 17.3% of the 289 normal-weight subjects (*p* < 0.001). The same trend was observed in men and women. Abdominal obesity (high WC) was significantly associated with low HDL-C.

**Table 2:** Serum lipid and body composition according to HDL-C level. LDL-C, low-density lipoprotein cholesterol.

	*Men*				*Women*			
	All (*n* = 271)	Low HDL-C (*n* = 61)	Normal HDL-C (*n* = 210)	*p* ^1^	All (*n* = 270)	Low HDL-C (*n* = 88)	Normal HDL-C (*n* = 182)	*p*
Ratio TC/HDL-C	3.37 ± 1.26	4.46 ± 1.53	3.05 ± 0.96	<0.001	3.21 ± 1.06	4.13 ± 1.10	2.76 ± 0.69	<0.001
LDL-C	2.53 ± 0.98	2.52 ± 1.01	2.53 ± 0.98	0.91	2.64 ± 0.91	2.78 ± 0.88	2.57 ± 0.93	0.085
Triglycerides mmol/L	0.81 ± 0.46	0.89 ± 0.42	0.79 ± 0.46	0.14	0.69 ± 0.33	0.82 ± 0.34	0.63 ± 0.30	0.001
Body Mass Index	22.3 ± 3.8	23.4 ± 4.2	21.9 ± 3.6	0.008	26.0 ± 6.1	28.2 ± 7.2	25.0 ± 5.2	<0.001
Waist circumference	82.2 ± 10.4	84.9 ± 11.3	81.4 ± 10.1	0.022	88.0 ± 13.7	92.6 ± 15.2	85.7 ± 12.3	<0.001
Fat-free mass kg	55.6 ± 7.7	56.7 ± 8.6	55.2 ± 7.4	0.18	44.6 ± 6.3	47.0 ± 7.3	43.5 ± 5.4	<0.001
Fat mass kg	11.4 ± 6.6	12.6 ± 6.7	11.1 ± 6.6	0.11	22.2 ± 11.7	26.0 ± 13.8	20.4 ± 10.0	<0.001

^1^ *t*-test.

**Table 3:** Nutritional status, diet quality and lifestyle according to HDL-C level.

	*Women*					*Men*			
	(*n*)	Low HDL-C (%)	Normal HDL-C (%)	*p* ^1^		(*n*)	Low HDL-C (%)	Normal HDL-C (%)	*p*
*BMI status* ^2^				< *0.001*					<0.001
Underweight	(27)	33.3	66.7			(34)	29.4	70.6	
Normal weight	(106)	18.9	81.1			(183)	16.4	83.6	
Overweight/ obese	(137)	43.1	56.9			(54)	38.9	61.1	
*WC status* ^3^				*0.023*					0.045
Abdominal obesity	(187)	36.9	63.1			(37)	35.1	64.9	
Normal WC	(83)	22.9	77.1			(234)	20.5	79.5	
*Micronutrient intake adequacy*				*0.10*					0.04
1st tertile	(113)	34.5	65.5			(83)	31.3	68.7	
2nd tertile	(93)	37.6	62.4			(76)	22.4	77.6	
3rd tertile	(64)	21.9	78.1			(112)	16.1	83.9	
*Preventive diet score*				*0.35*					0.95
1st tertile	(75)	37.3	62.7			(74)	23.0	77.0	
2nd tertile	(133)	33.1	66.9			(126)	23.0	77.0	
3rd tertile	(62)	25.8	74.2			(71)	21.1	78.9	
*Physical activity* ^4^				< *0.001*					0.65
Inactive	(69)	50.7	49.3			(27)	25.9	74.1	
Active	(201)	26.4	73.6			(244)	22.1	77.9	
*Alcohol consumption*				*0.08*					0.67
None	(171)	31.0	69.0			(93)	21.5	78.5	
Moderate	(72)	29.2	70.8			(97)	20.6	79.4	
High	(27)	51.9	48.1			(81)	25.9	74.1	

^1^ Chi^2^; ^2^ Underweight: BMI < 18.5; normal weight: BMI 18.5–24.9; overweight/obese: BMI ≥ 25; ^3^ Abdominal obesity: WC ≥ 80 cm in men, ≥94 cm in women; ^4^ Active: ≥half-hour/day of moderate or vigorous physical activity.

Regarding dietary quality, a higher micronutrient adequacy score was associated with a lower rate of low HDL-C in women; the trend was not significant in men. The preventive diet score showed no relationship with HDL-C. The proportion of low HDL-C was significantly lower among the physically active than inactive men. In women, physical activity did not appear to be significantly related to HDL-C. A higher consumption of alcohol tended to be associated with lower HDL-C, but only in men.

Mean percent intake adequacy for individual micronutrients included in the micronutrient score is displayed in Table 4 according to HDL-C level. Intakes of vitamin A, niacin, vitamin B_12_, zinc and calcium were significantly better in those with normal HDL-C level, thus explaining the higher micronutrient scores of the latter compared to subjects with low HDL-C. Of note, percent adequacy was lowest for vitamin B_12_. When the data were analyzed by sex, micronutrient adequacy according to HDL-C level was significant in both men and women for zinc. In women, adequacy percentages for vitamin A, vitamin E and vitamin B_12_ adequacy were also significant, while in men, vitamin C and calcium were (data not shown).

**Table 4:** Micronutrient intake adequacy % according to HDL-C level.

	All	Low HDL-C	Normal HDL-C	*p* ^1^
Vitamin A	99.5 ± 4.3	98.8 ± 6.9	99.7 ± 2.7	0.019
Vitamin E	73.3 ± 22.5	72.6 ± 23.2	73.6 ± 22.1	0.640
Vitamin C	99.8 ± 2.3	99.6 ± 3.3	99.9 ± 1.7	0.203
Thiamine	99.0 ± 4.3	98.5 ± 5.2	99.2 ± 3.9	0.099
Riboflavin	95.2 ± 10.6	94.4 ± 11.3	95.4 ± 10.3	0.319
Niacin	97.6 ± 7.5	96.3 ± 9.2	98.0 ± 6.7	0.017
Vitamin B_12_	66.4 ± 26.6	61.7 ± 25.6	68.2 ± 26.7	0.011
Vitamin B_6_	99.8 ± 1.8	99.9 ± 1.8	99.8 ± 1.9	0.808
Folate	94.6 ± 11.8	94.0 ± 12.1	94.8 ± 11.7	0.497
Pantothenic acid	95.8 ± 9.5	94.9 ± 10.1	96.1 ± 9.2	0.193
Magnesium	100 ± 0.29	100 ± 0.0	100 ± 0.34	0.538
Zinc	87.6 ± 15.8	84.3 ± 16.5	88.8 ± 15.4	0.003
Iron	86.4 ± 20.2	83.9 ± 21.7	87.3 ± 19.6	0.084
Calcium	84.5 ± 22.4	79.4 ± 24.3	86.4 ± 21.4	0.001
Micronutrient adequacy score (maximum 14)	10.0 ± 2.7	9.4 ± 2.7	10.2 ± 2.6	0.001

^1^ *t* test.

### 3.4. Diet and Lifestyle Determinants of HDL-C

The multiple linear regression model of HDL-C on lifestyle and dietary factors controlling for socio-demographic variables (Table 5) showed that a higher micronutrient adequacy score was independently and positively associated with higher HDL-C. Micronutrient adequacy was also significantly and negatively associated with the ratio of TC/HDL-C when the latter was substituted for HDL-C as a dependent variable (data not shown). The preventive diet score and other lifestyle parameters were not significant predictors of HDL-C (or TC/HDL-C). Tobacco smoking was not included in the models, because very few men smoked and women did not smoke. WC tended to be negatively associated with HDL-C, while being significantly and positively associated with TC/HDL-C (data not shown). BMI was not significant in this model, since its relationship with HDL-C was not linear. Sex, age and residence area were significant determinants, but there was no interaction of sex and location.

**Table 5:** Linear regression of HDL-C on dietary and lifestyle factors controlling for socio-economics.

	*HDL-C*	
	β (standardized)	*p*
Sex (0 = M; 1 = F)	0.18	0.015
Age years	0.12	0.01
Zone (0 = rural; 1 = semi-urban; 2 = urban)	−0.145	0.025
Education (0 = none; 1 = primary; 2 = secondary+)	0.045	0.37
Household amenity score tertile	−0.041	0.37
Alcohol consumption (g/day)	0.025	0.57
Physical activity (0 = None; 1 ≥ 30 min/day)	0.086	0.08
Micronutrient intake adequacy score (0–14)	0.125	0.01
Preventive diet score (0–8)	−0.004	0.91
BMI	−0.009	0.70
Waist circumference	−0.165	0.10
Interaction sex by zone	0.046	0.59
*R*^2^ of model	0.130	<0.001

### 3.5. Association of HDL-C with Other Cardiometabolic Risk Factors

Table 6 shows the proportion of men and women exhibiting CMRF according to their HDL-C status and the odds ratios (OR) for individual and clustered CMRF in low HDL-C groups. The CMRF considered are those of the MetS, and we added hyperhomocysteinaemia and TC/HDL-C. The odds ratio (OR) for insulin resistance was significant in women, with a higher rate in subjects with low HDL-C; a similar trend was observed in men, but it was not significant. The ORs for elevated TC/HDL-C ratio were significant in men and women, with a several-fold increased likelihood of a high ratio when HDL-C was low. Even though high triglyceride concentrations were not highly prevalent in this population, we observed in women an increased rate with low HDL-C. The other individual CMRF examined in the study, that is, high blood pressure, high serum homocysteine and elevated fasting glycaemia, were not significantly associated with HDL-C. The OR for the MetS in subjects with low HDL-C was significant in women, but not in men. When only the MetS risk factors other than low HDL-C were combined (high blood pressure; elevated fasting glycaemia; high triglyceride concentration; abdominal obesity), the likelihood of clustering of at least two risk factors was not significantly higher in low HDL-C men or women.

**Table 6:** Prevalence and odds of cardio-metabolic risk factors in low HDL-C subjects.

	*Men*			*Women*		
	Low HDL-C (%)	Normal HDL-C (%)	OR (95% CI) ^1^	Low HDL-C (%)	Normal HDL-C (%)	OR (95% CI) ^1^
High blood pressure	14.8	25.7	0.50 (0.23–1.08)	23.9	25.8	0.90 (0.50–1.63)
Insulin resistance	21.3	15.2	1.51 (0.73–3.09)	46.6	27.5	**2.30 (1.36–3.91)**
Hyperhomocysteinaemia	44.3	54.6	0.66 (0.37–1.17)	27.6	23.3	1.25 (0.70–2.24)
Elevated fasting glycaemia	8.2	10.0	0.80 (0.29–2.23)	8.0	9.3	0.84 (0.33–2.10)
High ratio TC/HDL-C	26.2	4.8	**7.11 (3.03–16.70) ^2^**	52.3	4.4	**23.82 (10.5–54.2)**
High triglyceride concentration	3.3	3.3	0.98 (0.20–4.86)	2.3	0.0	^3^
Abdominal obesity	21.3	11.4	2.10 (0.996–4.42)	78.4	64.8	**1.97 (1.09–3.56)**
MetS ^4^	9.8	1.4	2.4 (0.94–5.98)	28.4	3.3	**3.8 (1.85–7.84)**
≥2 MetS components other than low HDL-C	14.8	10.5	1.48 (0.64–3.41)	28.4	25.3	1.17 (0.66–2.08)

^1^ OR = odds ratio for the risk factor(s) in low HDL-C subjects; CI = confidence intervals; ^2^ Significant (*p* < 0.05) values in bold; ^3^ cannot be computed; ^4^ MetS = metabolic syndrome. TC = total cholesterol.

## 4. Discussion

The Benin study on nutrition transition and CMRF reported previously that the main components of the MetS present in the Benin population were high blood pressure, low HDL-C and abdominal obesity [20], in accordance with other studies in West Africa [17] and in black South Africans [16].

### 4.1. Low HDL-C at Both Ends of the BMI Continuum?

We found that both underweight and overweight as nutritional status indicators tended to be associated with low HDL-C. The rate of low HDL-C according to BMI status indeed showed a J-curve. This suggests that chronic energy deficiency reflected in low BMI and not only overweight may be associated with enhanced cardio-metabolic risk, because of low HDL-C. The evidence of an inverse relationship between HDL-C and cardio-metabolic risk is robust. In a review, 31 out of 58 prospective studies showed a significant inverse association of HDL-C with CVD risk, even when controlling for other lipid factors [35]. In our study, low HDL-C was associated with higher odds for other CMRF, such as high TC/HDL-C ratio and insulin resistance (among women). 

Low HDL-C and other blood lipid alterations have been reported in malnourished children [36,37]. To our knowledge, ours is one of the first studies in adults to consider the dual burden of malnutrition, that is, the coexistence in the same individuals of undernutrition or micronutrient malnutrition and diet-related chronic disease risk factors. The double burden of malnutrition has usually been examined at population or household level, rather than in individuals [38]. Furthermore, the more commonly described individual phenotype of dual burden is the coexistence of overweight and micronutrient malnutrition [39], or else, of stunted height and hypertension [40], not current underweight and chronic disease risk. Undernutrition in early life—*in utero* or in infancy—has also been associated with subsequent elevated risk for CVD, diabetes and abdominal obesity [41]; this represents the intergenerational form of the double nutritional burden [42].

We describe in this paper a phenotype of concurrent underweight and high-risk blood cholesterol profile (low HDL-C and high TC/HDL-C) in Benin. In Tanzania, lower HDL-C was reported in rural adults compared to city dwellers, whereas the other serum lipid components appeared less deteriorated in the rural subjects [43]. This is consistent with the association of low HDL-C with underweight, since it is more highly prevalent in rural areas of developing countries, as we also observed [24].

However, the significance and metabolic implications of low HDL-C in underweight *vs*. overweight individuals cannot be ascertained in this study. Further research is required to examine not only HDL-C and other cardio-metabolic risk factors in adults exposed to under- and over-nutrition, but also the functionality of HDL-C.

### 4.2. Micronutrient Intake Independently Associated with HDL-C

A major finding of our study is the observed independent association of lower micronutrient intake adequacy with lower HDL-C, when controlling for anthropometric status, socio-economic and demographic factors, physical activity and alcohol consumption, although only 13% of the variance of HDL-C was explained by the full regression model. Dietary data was obtained for two or three 24-h food recalls on non-consecutive days using the multiple pass method [44] for optimal quality of the data. In our study, the micronutrient adequacy score was consistently protective, whereas the preventive diet score was not, but this may be ascribed to the score itself or to local diets, which were not highly atherogenic even in the large city [25]. Micronutrient deficiencies are suspected to increase the risk of chronic diseases, and so far, there is some evidence in humans primarily for folate, vitamin B_12_, zinc and iron [45,46,47,48]. In our study, the micronutrient adequacy score, which included 14 vitamins and minerals, was positively associated with HDL-C level. Intake adequacy of five vitamins, including B_12_, but not folate, was significant. We also observed that intake adequacy of zinc and calcium was significant. This further suggests that undernutrition, whether general or micronutrient-specific, and not only overnutrition, increases cardio-metabolic risk. In our study in Burkina Faso [21,43], the double burden phenotypes combining micronutrient deficiencies and CMRF other than obesity were associated with dietary patterns characterized by low adequacy of vitamins B_12_ and B_6_, but not zinc [49]. Vitamin D deficiency has also been found to be related to lower HDL-C in women [50]. The observed associations between micronutrient deficiencies and HDL-C do not disclose putative biological mechanisms that could explain the links. However, the connection of micronutrients with chronic diseases has been a subject of research and hypotheses for several years [51].

HDL-C may decline as a result of poor nutrition (or low fat intake like in the vegetarians) and also because of inflammation and hyperhomocysteinaemia. While there is limited information, inflammation may be widespread and contribute to chronic disease in Africa [52]. In Benin, elevated homocysteine was highly prevalent (52.2% of men, 24.7% of women) [53], but we found no independent association with HDL-C. Even if HDL-C is high, its functionality may be altered in certain cardio-metabolic conditions, in which case HDL-C may not then fully play its protective role [54,55].

### 4.3. Low HDL-C and High TC/HDL-C as Markers of at-Risk Lipid Profile in Sub-Saharan Africans

In our study, the overall prevalence of high TC/HDL-C was also high, although not as high as that of low HDL-C. Since high LDL-C was not frequent (7.8%; no difference between men and women), high ratios are likely ascribable to low HDL-C rather than elevated LDL-C. The odds for other CMRF in low-HDL-C subjects was indeed highest for elevated TC/HDL-C. In populations at risk of CVD because of low HDL-C, it was reported that TC/HDL-C ratio would identify more at-risk subjects than Framingham risk tables [56]. This suggests that TC/HDL-C could serve as single marker of an at-risk lipid profile in sub-Saharan Africans.

The odds for insulin resistance were also significantly increased in low HDL-C subjects, at least in women, compared with individuals with normal HDL-C. However, no differences were detected in the occurrence of high blood pressure, hyperglycemia or hyperhomocysteinaemia. It would be tempting to speculate on the link between higher insulin resistance and inadequate zinc intakes, since both were observed in low HDL-C subjects in our study. Experimental studies in rats have shown that maternal zinc deficiency results in insulin resistance in the offspring [57], and the connection between zinc, the metabolic syndrome and diabetes is documented in humans [48].

### 4.4. Strengths and Limitations

Among the strengths of the study, we will mention the collection of extensive data on diet and lifestyle, in addition to being one of the first studies to examine both ends of the nutrition continuum in sub-Saharan African adults. The cross-sectional nature of the study is undoubtedly a limitation. Another limitation is that overweight and obese individuals had to be collapsed into a single group owing to small number of obese men. Regarding abdominal obesity, the WC cut-points recently proposed as predictors of MetS in black South Africans [58] may be relevant in further studies on cardiometabolic risk factors in sub-Saharan Africa, as ethno-specific WC cut-offs are advocated [6], but do not as yet exist for Africans.

## 5. Conclusions

The study on anthropometric nutritional status, diets and lifestyles and cardio-metabolic risk factors in apparently healthy Benin adults suggests that low HDL-C may also be associated with underweight and not only overweight/obesity. Additionally, poor micronutrient intakes, notably vitamin B_12_ and zinc, were significant and independent predictors of low HDL-C. A high ratio of TC/HDLC was strongly associated with low HDL-C, and it could be worth further assessing its value as a marker of cardiometabolic risk in sub-Saharan populations. The findings of this study will have to be confirmed in longitudinal studies, but they strongly suggest that in order to tackle diet-related chronic diseases in nutrition transitioning countries, the double burden of malnutrition needs to be addressed, that is, not only obesity, but also underweight and poor micronutrient nutrition.
